# Data of added-value lipid production, Arachidonic acid, among other lipids by *Mortierella elongata*, using low cost simulated wastewater

**DOI:** 10.1016/j.dib.2017.07.015

**Published:** 2017-07-14

**Authors:** Bruna Soares Fernandes, Márcia Cristina Fernandes Messias, Patrícia de Oliveira Carvalho, Marcelo Zaiat, José Geraldo da Cruz Pradella

**Affiliations:** a Brazilian Bioethanol Science and Technology Laboratory (CTBE), Brazilian Centre of Research in Energy and Materials (CNPEM), Campinas, SP, Brazil; b Multidisciplinary Research Laboratory, San Francisco University (USF), Bragança Paulista, SP, Brazil; c Biological Processes Laboratory, Center for Research, Development and Innovation in Environmental Engineering, São Carlos School of Engineering (EESC), University of São Paulo (USP), São Carlos, SP, Brazil

**Keywords:** Arachidonic acid (ARA), Lipids, *Mortierella elongata*, Polyunsaturated Fatty Acids (PUFA), Sugarcane wastewater- vinasse

## Abstract

This article presented an innovative data of feasibility to produce Arachidonic acid (ARA), as added-value Polyunsaturated Fatty Acids (PUFA), among other lipids from *Mortierella elongata*, using simulated low cost sugarcane wastewater, vinasse, as a carbon source. Data from lipids quantification by total lipids extraction and by lipid classes was presented. *M. elongata* was able to produce 156.45mg of ARA per g of total lipids.

**Specifications Table**

**Table:** 

Subject area	*Biology*
More specific subject area	*Lipids production by biotechnological process and their extraction*
Type of data	*Table, text file, graph, figure*
How data was acquired	*Gas Chromatography - Flame Ionization Detector (GC-FID)*
Data format	*Raw data collection and analysis*
Experimental factors	*Biomass production and oven drying and lipids extraction, classification, transesterification and analyzed in the GC-FID.*
Experimental features	*The microorganism was cultivated in low cost simulated sugarcane wastewater, vinasse.*
Data source location	*Mortierella elongata (CBS 121.71) was acquired from the CBS-KNAW Fungal Biodiversity Centre, Netherlands. All experiments were developed in Campinas-SP, Brazil.*
Data accessibility	*Data are presented in this article*

**Value of the data**

• The obtained data are innovative and important in the research area of biotechnology providing results of lipids production by *M. elongata*, a fungus, using low cost substrate, sugarcane wastewater vinasse, applied in Nutrition and Pharmaceutical Industry.
• Sugarcane vinasse, the main residue of ethanol process production, is a promising low cost carbon source and produced in large amounts. Since there is estimative of a growth in Brazilian domestic ethanol consumption of 58.8 billion liters by 2019 [1], with a corresponding vinasse production of 588 billion liters.
• The data can be most useful for the researcher, research student, academician and industry to acquire innovative process development and production.
• The described data identify a sustainable mechanism and with low cost to produce added-value lipid mainly, Arachidonic acid, since it is recommend injection of eicosapentaenoic acid (EPA), docosahexaenoic acid (DHA) and Arachidonic acid (ARA) in the infant [2], and for adults prevent coronary heart disease [3] and cancer [4].

## 1 Data

*Mortierella elongata* is an oleaginous fungus able to accumulate lipids. The data of lipids extraction from *M. elongata* are presented in the Table 1. The total lipids extraction from *M. elongata* were 14.95% and 8.54%, by MC and HIP method, respectively, using simulated low cost sugarcane wastewater, vinasse as a carbon source. Triacylglycerol (TGA) was highest lipid class observed, which is in accordance with previous studies for genus *Mortierella*, using other substrate [5]. Considering the HIP and MC lipids extraction process, the mixture Hexane/Isopropanol has less polar lipid interaction properties than Chloroform/Methanol [6], which reduce the extraction of phospholipids as observed in the data presented in the Table 1.

**Table 1:** Lipids classes in percentage extracted from *Mortierella elongata.*

**Average of Lipids (%) (w:w)**	**Method**	
	**MC**	**HIP**
**TAG (% of TL)**	71.87%	92.54%
**NEFA (% of TL)**	25.74%	4.68%
**PP (% o TL)**	2.43%	2.78%
**TOTAL LIPIDS (% of DW)**	14.95%	8.57%

TAG – triacylglycerol, NEFA – free fatty acids, PP –phospholipids, TL – total lipids, DW – dry weight.

**Fig. 1:**
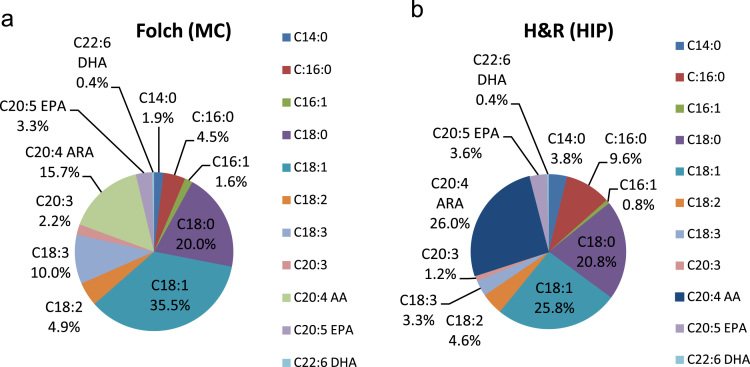
Main fatty acids profile of *Mortiella elongata* by a) Folch and b) Hara and Radin lipid extraction methods.

**Fig. 2:**
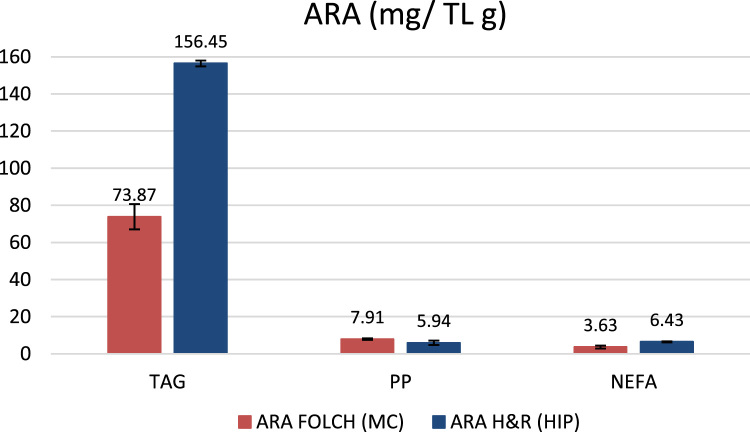
Arachidonic acid (ARA) concentration by lipids extraction method.

According to Fig. 1 (Chromatograms in the Supplementary data), it is presented the data profile of lipids by chain length and saturation, since C18:0, C18:1 and C20:4, the Arachidonic acid (ARA) were the predominant lipids. The Fig. 2 shows data of percentage and amount of ARA extracted by lipid class. In HIP method there was a high ARA extraction by percentage (26.0 %) of total fatty acids and in recovery weight (156.45mg of ARA g-1 of TGA and 168.81mg of ARA g-1 of TL) in five days of culture (Fig. 2), using simulated vinasse as a carbon source.

## 2 Experimental design, materials and methods

### 2.1 Microorganism cultivation

*Mortierella elongata* (CBS 121.71) was inoculated per 5 days, at 29 °C, in shaker at 200rpm, with culture medium simulating the main carbon sources of sugarcane vinasse [7], main sub-product of ethanol process production.

### 2.2 Simulated vinasse composition

Simulated sugarcane vinasse composition, based on the real wastewater characterization: (g/L): sucrose 10, glucose 6, glycerol 6, lactic acid 3, acetic acid 1, Yeast Extract 1, and 10% (v:v) of trace elements stock solution – (g/L): KH2PO4 0.65; (NH4)2SO4 14.00; MgSO4.7H2O 3.00; CaCl2.2H2O 39.97; FeSO4. 7H2O 0.05; ZnSO4.7H2O 0.013; MnSO4.H2O 0.016; CoCl2 0.02; NaNO3 28.00;KOH 20.00.

### 2.3 Lipids extraction

The oven dried biomass of *Mortierella elongata* (Dry Weight- DW) (80°C) was macerated and disrupted using HCl 1%, and the total lipids was extracted by Folch et al. [8] method, using Chloroform/Methanol (2:1) (CM) as solvents, and by Hara and Radin [9] method, using Hexane/Isopropanol (2:3) (HIP) as solvents. The total lipid (TL) was stratified by classes (phospholipids – PP, triacylglycerol - TAG, non-esterified fatty acids or free fatty acids – NEFA) using Solid-phase extraction (SPE) protocol [10].

### 2.4 Lipids quantification

The stratified total lipids and by class were transesterified with NaOH-methanol, Boron Trifluoride, and Hexane. The Fatty Acids Methyl Ester (FAME) were analyzed in the GC-FID (Chrompack 9001) using CP-Sil 88 capillary column (Chromatograms in the Supplementary data).

## Footnotes

Appendix A Supplementary data associated with this article can be found in the online version at doi:10.1016/j.dib.2017.07.015.
