# Supplementary material for: Data of added-value lipid production, Arachidonic acid, among other lipids by Mortierella elongata, using low cost simulated wastewater
Source: Data Brief. 2017 Jul 14;14:255–9. doi: 10.1016/j.dib.2017.07.015 (PMC5567393; doi:10.1016/j.dib.2017.07.015)
Supplement: Supplementary file 2 — Supplementary material [file mmc2.docx]

Supplementary data - *Data of added-value lipid production, Arachidonic acid, among other lipids by Mortierella elongata, using low cost simulated wastewater*

**SUPELCO 37 100%**

**Peak No. Peak ID Ret Time Height Area Conc.**

1 2,605 16,447 631,646 0,0036

2 3,600 65533,801 -3526013,500 -19,9608

3 4,205 83400,109 266753,344 1,5101

4 4,947 98482,484 894309,250 5,0627

5 5,660 44279,652 531936,625 3,0113

6 6,658 91338,703 1276016,000 7,2235

7 7,853 42209,688 679698,313 3,8478

8 9,235 92472,086 1529085,000 8,6562

9 10,342 48197,379 547832,563 3,1013

10 10,620 60074,711 964431,313 5,4596

11 11,403 1550,405 27062,447 0,1532

12 11,765 49484,949 527945,500 2,9887

13 12,078 150259,563 2387210,750 13,5140

14 12,978 53800,820 709822,688 4,0183

15 13,440 40348,691 590536,438 3,3430

16 14,265 59680,973 670904,375 3,7980

17 14,720 107347,547 1074233,500 6,0812

18 15,112 59725,879 503216,000 2,8487

19 15,302 131387,578 1177111,875 6,6636

20 15,790 43460,684 396578,875 2,2450

21 16,218 59555,098 501384,156 2,8383

22 16,787 111737,742 617526,750 3,4958

23 16,888 65039,844 437236,531 2,4752

24 17,310 106778,977 771191,188 4,3657

25 17,800 54001,824 398874,438 2,2580

26 18,267 51292,164 416088,375 2,3555

27 19,017 60802,465 415509,000 2,3522

28 19,123 39066,008 279701,125 1,5834

29 19,495 3506,529 38651,977 0,2188

30 19,740 44454,477 395969,063 2,2416

31 19,903 34578,016 278323,813 1,5756

32 20,548 26221,422 242666,172 1,3737

33 21,237 24950,221 231193,672 1,3088

34 21,643 2161,943 22075,387 0,1250

35 21,915 18158,018 171717,063 0,9721

36 22,533 30543,217 427355,438 2,4193

37 23,600 18498,734 223758,500 1,2667

38 24,508 2089,177 39908,414 0,2259

39 24,740 2651,455 49029,563 0,2776

40 25,117 1847,355 59533,859 0,3370

41 25,803 1594,308 61417,988 0,3477

42 26,510 1335,127 38300,809 0,2168

43 27,053 13475,336 98609,961 0,5582

44 27,657 1581,130 65023,949 0,3681

45 29,238 548,941 15586,193 0,0882

46 29,665 4200,440 130805,953 0,7405

47 31,757 688,935 7973,694 0,0451

HIP1-total lipids

Peak No. Peak ID Ret Time Height Area Conc.

1 3,458 970446,313 13643302,000 66,1869

2 4,362 1592,890 25813,662 0,1252

3 4,785 885,359 17932,357 0,0870

4 5,342 372,969 4135,784 0,0201

5 5,532 352,062 3386,112 0,0164

6 5,972 85,676 579,426 0,0028

7 6,298 69,821 796,200 0,0039

8 6,818 293,246 2107,871 0,0102

9 7,238 867,466 25839,557 0,1254

10 7,740 434,260 5658,216 0,0274

11 8,770 17612,666 75341,188 0,3655

12 9,030 400,028 2499,484 0,0121

13 9,260 191,819 1143,583 0,0055

14 9,573 135,452 925,100 0,0045

15 9,885 4322,813 17882,109 0,0868

16 11,092 239268,188 1089036,250 5,2832

17 11,878 2678,297 26837,320 0,1302

18 12,288 7355,163 35225,660 0,1709

19 12,863 506,374 5898,962 0,0286

20 13,045 1782,413 11837,980 0,0574

21 13,577 192232,250 963142,563 4,6724

22 14,025 2905,833 25297,510 0,1227

23 14,298 419378,375 130323,031 0,6322

24 16,010 9534,936 93862,555 0,4553

25 16,160 43914,188 419573,219 2,0354

26 19,732 178984,516 1659904,875 8,0526

27 14,780 302,472 2404,704 0,0117

28 15,342 84753,109 346575,375 1,6813

29 16,160 43173,652 222568,453 1,0797

30 16,682 11953,619 72381,914 0,3511

31 17,612 993,565 6841,019 0,0332

32 17,890 1868,047 13862,687 0,0673

33 18,368 507,591 3221,353 0,0156

34 18,870 31683,260 249178,422 1,2088

35 19,402 620,089 5552,012 0,0269

36 19,732 178561,844 1001988,063 4,8609

37 20,440 389,614 3531,443 0,0171

38 20,670 487,838 4672,680 0,0227

39 20,942 468,285 6973,802 0,0338

40 21,383 556,633 4079,774 0,0198

41 21,692 3509,273 23638,887 0,1147

42 22,067 1327,268 16471,846 0,0799

43 22,335 14868,699 162898,703 0,7903

44 23,398 13052,356 127999,953 0,6210

45 24,630 251,908 2709,275 0,0131

46 24,930 93,506 1229,923 0,0060

47 25,690 34,681 450,749 0,0022

48 27,430 1106,703 16804,234 0,0815

49 28,107 130,604 1833,061 0,0089

50 28,920 613,090 17619,787 0,0855

51 30,190 470,533 5196,866 0,0252

52 30,588 84,241 837,752 0,0041

53 30,962 119,840 1110,874 0,0054

54 31,378 121,627 2378,306 0,0115

HIP.2

Peak No. Peak ID Ret Time Height Area Conc.

1 3,490 970244,688 12689727,000 62,9533

2 4,483 667,382 3943,300 0,0196

3 4,765 397,819 3327,921 0,0165

4 5,512 642,514 2957,599 0,0147

5 5,937 111,939 821,224 0,0041

6 6,263 431,697 4042,252 0,0201

7 6,597 134,160 492,604 0,0024

8 7,202 1197,269 36641,816 0,1818

9 7,702 496,396 5260,972 0,0261

10 8,292 53,840 189,401 0,0009

11 8,732 19742,578 89528,922 0,4441

12 8,990 510,750 5645,220 0,0280

13 9,557 218,103 1581,309 0,0078

14 9,850 4799,055 24281,492 0,1205

15 10,560 97,764 847,970 0,0042

16 11,068 264081,188 1277202,000 6,3362

17 11,848 3225,643 33764,922 0,1675

18 12,260 7716,838 40701,520 0,2019

19 12,835 541,675 5637,013 0,0280

20 13,013 1948,149 13514,588 0,0670

21 13,542 188451,219 1004691,625 4,9842

22 14,262 438713,875 2265339,250 11,2383

23 15,295 89029,602 416507,344 2,0663

24 15,968 9387,514 51462,508 0,2553

25 16,113 46617,305 217312,422 1,0781

26 16,637 12739,743 -1455758,750 -7,2220

27 19,655 183077,641 1776111,500 8,8112

28 17,140 92,178 319,099 0,0016

29 17,555 1107,901 7707,350 0,0382

30 17,833 1779,416 13278,509 0,0659

31 18,313 360,503 1804,236 0,0090

32 18,805 32990,641 243898,313 1,2100

33 19,328 446,402 3128,772 0,0155

34 19,655 182484,500 1000848,188 4,9652

35 20,368 207,693 1237,298 0,0061

36 20,597 333,445 2126,768 0,0106

37 20,867 402,611 4834,345 0,0240

38 21,308 445,588 3255,291 0,0161

39 21,620 3496,161 22274,217 0,1105

40 21,993 1113,822 13855,350 0,0687

41 22,272 14721,291 154696,438 0,7674

42 23,337 12693,421 116655,641 0,5787

43 24,032 238,005 3657,540 0,0181

44 24,555 216,753 2478,264 0,0123

45 24,868 86,397 994,247 0,0049

46 25,412 37,995 194,900 0,0010

47 25,762 22,661 92,798 0,0005

48 26,993 38,002 273,028 0,0014

49 27,365 1130,925 18554,908 0,0921

50 28,452 136,535 2549,636 0,0126

51 28,828 595,485 12622,593 0,0626

52 30,055 431,819 5143,427 0,0255

53 30,557 90,410 1064,088 0,0053

54 30,872 115,037 1272,783 0,0063

55 31,265 131,070 2784,512 0,0138

FOLCH.2

Peak No. Peak ID Ret Time Height Area Conc.

1 0,190 26,615 136,701 0,0005

2 3,458 970810,438 14441583,000 56,7549

3 3,960 2539,829 16412,707 0,0645

4 4,383 1590,328 11075,397 0,0435

5 4,687 999,000 8584,501 0,0337

6 5,415 143,970 469,666 0,0018

7 5,520 84,649 310,496 0,0012

8 5,827 107,050 831,702 0,0033

9 6,150 562,269 4871,343 0,0191

10 6,645 2739,751 129816,328 0,5102

11 7,568 961,712 16968,895 0,0667

12 7,980 419,375 7333,395 0,0288

13 8,597 27688,992 129328,359 0,5083

14 8,853 784,028 6735,528 0,0265

15 9,100 424,859 3840,764 0,0151

16 9,420 149,290 1246,687 0,0049

17 9,713 6790,683 29435,180 0,1157

18 10,025 194,716 2036,315 0,0080

19 10,415 117,205 704,797 0,0028

20 10,953 355267,500 1715408,750 6,7415

21 12,138 10481,835 43202,547 0,1698

22 12,707 531,546 3010,194 0,0118

23 12,892 2524,587 12111,966 0,0476

24 13,450 282266,531 1516041,875 5,9580

25 14,165 621155,438 3461532,750 13,6037

26 14,920 3533,563 27373,492 0,1076

27 15,172 149560,109 588618,063 2,3132

28 15,823 16913,424 80792,375 0,3175

29 15,968 78689,969 300026,813 1,1791

30 16,480 21195,729 120457,094 0,4734

31 17,383 1463,460 8300,704 0,0326

32 17,658 3128,719 20601,609 0,0810

33 18,118 620,339 2515,500 0,0099

34 18,605 58464,191 413889,188 1,6266

35 19,120 833,401 6558,030 0,0258

36 19,477 298430,813 1689804,000 6,6409

37 20,165 359,844 2424,907 0,0095

38 20,390 675,266 10495,277 0,0412

39 21,112 722,558 4554,262 0,0179

40 21,425 5814,975 36036,355 0,1416

41 21,790 2055,929 25723,143 0,1011

42 22,125 23625,867 260526,422 1,0239

43 23,183 20481,582 215466,844 0,8468

44 24,415 355,426 5850,195 0,0230

45 25,325 84,466 312,346 0,0012

46 25,653 107,981 2088,031 0,0082

47 26,295 77,083 787,463 0,0031

48 27,290 1685,129 26604,291 0,1046

49 27,823 166,192 2146,223 0,0084

50 28,478 167,337 2599,503 0,0102

51 28,793 913,354 18378,553 0,0722

52 30,037 621,427 6654,050 0,0262

53 30,523 118,970 1126,074 0,0044

54 30,772 180,366 582,989 0,0023

55 31,240 182,922 1201,999 0,0047

FOLCH.2

Peak No. Peak ID Ret Time Height Area Conc.

1 0,190 44,455 233,700 0,0008

2 3,448 970540,000 13483734,000 48,2332

3 3,932 2630,707 28301,301 0,1012

4 4,415 2176,800 16314,799 0,0584

5 4,732 1790,000 21436,098 0,0767

6 5,465 148,626 623,070 0,0022

7 5,582 91,102 379,053 0,0014

8 5,698 75,578 379,485 0,0014

9 6,182 938,408 12433,363 0,0445

10 6,715 2716,014 96569,711 0,3454

11 7,615 1024,973 14489,773 0,0518

12 8,065 377,952 6826,799 0,0244

13 8,648 37548,332 198623,422 0,7105

14 8,898 1039,211 7403,013 0,0265

15 9,132 583,163 4035,584 0,0144

16 9,515 158,442 999,531 0,0036

17 9,748 8459,395 45126,723 0,1614

18 10,065 259,973 2508,808 0,0090

19 10,465 189,389 988,600 0,0035

20 10,998 477289,750 2354388,250 8,4220

21 11,432 1315,373 12383,901 0,0443

22 11,632 4163,894 17897,000 0,0640

23 11,748 5089,947 21773,854 0,0779

24 12,148 12148,741 60033,102 0,2147

25 12,715 707,442 4026,711 0,0144

26 12,898 3097,263 16649,799 0,0596

27 13,482 321350,969 1914094,625 6,8470

28 13,898 4319,471 32853,672 0,1175

29 14,182 678557,000 4325614,000 15,4733

30 14,615 2388,137 13902,129 0,0497

31 14,748 1976,098 18628,184 0,0666

32 14,932 4592,919 33309,434 0,1192

33 15,182 196228,219 772209,188 2,7623

34 15,832 24802,406 112705,195 0,4032

35 15,965 101903,367 401424,688 1,4360

36 16,482 32000,592 413947,500 1,4807

37 16,982 109,000 350,000 0,0013

38 17,132 287,444 1072,500 0,0038

39 17,382 1964,000 12062,498 0,0431

40 17,665 4275,800 26354,693 0,0943

41 18,148 905,400 4029,999 0,0144

42 18,365 1700,854 7585,769 0,0271

43 18,648 76649,578 522682,594 1,8697

44 19,165 810,073 5973,120 0,0214

45 19,532 366290,719 2180025,000 7,7983

46 20,182 534,081 2668,174 0,0095

47 20,415 872,907 5191,142 0,0186

48 20,665 525,279 5737,857 0,0205

49 21,115 1039,882 5898,300 0,0211

50 21,432 8439,479 45499,051 0,1628

51 21,798 1583,543 17136,963 0,0613

52 22,132 36245,238 310925,563 1,1122

53 22,448 925,250 7569,522 0,0271

54 22,798 72,630 327,423 0,0012

55 23,165 32216,666 245870,594 0,8795

56 23,815 319,920 1911,360 0,0068

57 23,965 216,800 1334,840 0,0048

58 24,365 464,000 3806,500 0,0136

59 25,215 64,645 368,850 0,0013

60 25,565 62,194 543,246 0,0019

61 26,198 59,182 462,700 0,0017

62 27,165 2823,159 38926,699 0,1392

63 28,182 133,182 907,177 0,0032

64 28,298 136,273 448,025 0,0016

65 28,615 1445,662 20913,846 0,0748

66 29,832 855,074 7906,200 0,0283

67 30,632 97,588 807,000 0,0029

68 31,065 119,592 2012,779 0,0072

69 31,365 76,102 707,716 0,0025

**TAG 1**

**Peak No. Peak ID Ret Time Height Area Conc.**

1 0,032 75,429 661,100 0,0040

2 4,140 969744,438 9298867,000 55,6997

3 5,573 97,429 333,700 0,0020

4 6,207 2510,518 128849,602 0,7718

5 8,440 8642,696 49768,758 0,2981

6 8,740 443,804 5894,742 0,0353

7 9,423 120,016 1069,749 0,0064

8 9,823 2311,063 15717,450 0,0941

9 10,573 64,500 553,100 0,0033

10 11,240 144598,094 1036848,250 6,2107

11 11,957 2166,082 11914,675 0,0714

12 12,073 2741,476 27161,445 0,1627

13 12,440 6852,287 63109,672 0,3780

14 12,923 1271,492 14243,233 0,0853

15 13,073 2621,141 28720,660 0,1720

16 13,473 115582,211 841341,125 5,0396

17 13,857 5537,646 43770,238 0,2622

18 14,057 422150,188 2547234,750 15,2578

19 14,707 3934,661 35686,742 0,2138

20 14,907 64152,195 390152,938 2,3370

21 15,573 31174,305 239063,813 1,4320

22 15,990 10738,999 163728,141 0,9807

23 16,673 4706,737 41731,844 0,2500

24 16,890 4311,897 63640,992 0,3812

25 17,240 2294,080 27334,125 0,1637

26 17,423 2723,986 19569,686 0,1172

27 17,623 27161,520 210642,391 1,2617

28 18,007 2731,957 29177,684 0,1748

29 18,223 116972,117 690957,500 4,1388

30 18,757 2160,206 24122,273 0,1445

31 18,973 2290,367 22898,979 0,1372

32 19,123 2542,017 42026,809 0,2517

33 19,440 1917,944 12998,813 0,0779

34 19,657 3787,105 39119,094 0,2343

35 19,923 4272,149 53751,285 0,3220

36 20,173 7530,565 122212,289 0,7320

37 20,923 5753,814 115498,063 0,6918

38 21,373 3513,764 85600,594 0,5127

39 22,390 677,057 12939,634 0,0775

40 22,790 414,123 6153,545 0,0369

41 23,040 290,539 3916,577 0,0235

42 23,373 120,095 951,180 0,0057

43 23,873 413,943 4933,602 0,0296

44 24,073 48,486 247,500 0,0015

45 24,423 172,409 2327,932 0,0139

46 24,723 394,136 3593,469 0,0215

47 25,757 4014,104 41983,801 0,2515

48 26,723 2158,140 54613,199 0,3271

49 28,090 51,870 592,200 0,0035

50 28,857 523,636 9984,235 0,0598

51 29,290 246,333 3151,963 0,0189

52 30,123 120,610 2063,800 0,0124

53 31,840 70,552 1222,500 0,0073

**TAG2**

Peak No. Peak ID Ret Time Height Area Conc.

1 4,223 969493,438 11819402,000 69,0363

2 5,957 209,889 80,900 0,0005

3 6,573 816,600 12277,800 0,0717

4 7,173 379,304 1959,700 0,0114

5 8,323 9388,000 35883,699 0,2096

6 8,923 358,615 2000,300 0,0117

7 9,207 2175,500 7437,400 0,0434

8 10,007 186,813 1334,105 0,0078

9 10,190 148129,000 674829,500 3,9416

10 10,757 1234,714 4053,386 0,0237

11 10,857 1645,143 6243,115 0,0365

12 11,207 4977,818 23132,061 0,1351

13 11,707 346,545 2044,474 0,0119

14 11,857 1165,364 5282,168 0,0309

15 12,307 112948,656 627483,000 3,6651

16 12,723 2583,702 18429,141 0,1076

17 12,957 318512,156 1649262,875 9,6332

18 13,540 1670,832 13708,239 0,0801

19 13,690 1774,489 9220,203 0,0539

20 13,923 57790,953 258495,672 1,5099

21 14,690 41487,754 216680,719 1,2656

22 15,173 9116,983 73921,461 0,4318

23 15,907 2257,609 14541,191 0,0849

24 16,173 2668,564 30374,262 0,1774

25 16,540 724,877 5441,980 0,0318

26 16,757 859,152 5004,609 0,0292

27 16,973 33679,430 202176,438 1,1809

28 17,407 1611,979 13802,789 0,0806

29 17,673 184403,938 972533,813 5,6805

30 18,323 78,761 769,007 0,0045

31 18,473 992,375 5572,100 0,0325

32 18,673 458,875 2791,000 0,0163

33 19,273 4413,273 24856,752 0,1452

34 19,590 2846,636 29641,805 0,1731

35 19,923 6207,545 83720,227 0,4890

36 20,573 957,818 8345,010 0,0487

37 20,773 5400,364 56809,422 0,3318

38 21,190 2951,000 17904,639 0,1046

39 21,457 617,727 8145,147 0,0476

40 22,890 180,383 4006,800 0,0234

41 23,507 47,963 368,195 0,0022

42 24,090 219,426 2670,772 0,0156

43 24,890 468,360 7308,443 0,0427

44 25,307 485,809 9856,562 0,0576

45 25,623 235,151 2247,127 0,0131

46 25,973 6872,529 73429,461 0,4289

47 26,673 221,284 2544,498 0,0149

48 27,040 1526,680 53674,852 0,3135

49 28,523 177,281 3576,875 0,0209

50 29,423 306,000 7460,173 0,0436

51 29,990 293,429 3840,930 0,0224

52 31,090 164,355 3985,500 0,0233

**CE 1**

Peak No. Peak ID Ret Time Height Area Conc.

1 0,248 48,938 453,900 0,0008

2 3,107 970179,125 17493546,000 30,4163

3 3,648 4121,444 33839,898 0,0588

4 3,982 313,305 844,047 0,0015

5 4,082 3104,221 19420,199 0,0338

6 4,415 2807,274 33827,453 0,0588

7 4,915 357,579 1720,800 0,0030

8 5,132 319,667 1824,700 0,0032

9 5,515 5042,374 203602,469 0,3540

10 6,232 5499,044 48970,039 0,0851

11 7,082 2996,896 15349,100 0,0267

12 8,032 250031,547 1526261,125 2,6537

13 8,265 3126,556 13184,900 0,0229

14 8,482 901,636 3580,500 0,0062

15 8,682 349,143 2246,800 0,0039

16 9,065 35489,375 188107,094 0,3271

17 9,382 221,714 1369,928 0,0024

18 9,582 287,857 1602,885 0,0028

19 9,715 217,286 890,885 0,0015

20 9,932 139,625 860,100 0,0015

21 10,315 969272,875 9066114,000 15,7634

22 10,648 2137,933 13986,566 0,0243

23 10,832 18425,273 83532,469 0,1452

24 10,948 23163,400 108398,852 0,1885

25 11,365 29114,992 186444,813 0,3242

26 11,848 3225,800 15920,896 0,0277

27 12,048 8817,444 51923,801 0,0903

28 12,765 468496,500 5190991,000 9,0257

29 13,398 968106,375 11753845,000 20,4366

30 13,765 2496,055 15233,805 0,0265

31 14,032 6145,204 32321,664 0,0562

32 14,298 383657,000 1839882,625 3,1990

33 14,415 9005,454 29016,709 0,0505

34 15,032 255741,125 1245873,500 2,1662

35 15,282 934,209 5020,558 0,0087

36 15,532 91193,297 368976,688 0,6415

37 16,098 1595,826 8799,371 0,0153

38 16,265 4173,217 19859,676 0,0345

39 16,548 12711,982 56864,660 0,0989

40 16,915 3078,443 16935,855 0,0294

41 17,148 4153,191 18619,980 0,0324

42 17,382 200781,938 1240082,500 2,1562

43 17,848 1274,435 12711,954 0,0221

44 18,165 687491,875 4917723,500 8,5505

45 18,698 1655,730 10615,831 0,0185

46 18,865 3187,122 15907,198 0,0277

47 19,098 1118,870 7704,805 0,0134

48 19,215 786,243 5804,695 0,0101

49 19,465 3564,330 14569,048 0,0253

50 19,698 26100,078 103873,703 0,1806

51 20,015 2746,522 28003,156 0,0487

52 20,415 84434,664 655430,063 1,1396

53 20,898 969,496 7673,674 0,0133

54 21,232 85738,281 561391,313 0,9761

55 21,615 339,078 2491,066 0,0043

56 21,798 514,809 4856,779 0,0084

57 22,182 1338,609 8541,488 0,0149

58 22,898 188,217 3196,575 0,0056

59 23,165 379,391 6068,274 0,0106

60 23,582 229,913 1346,558 0,0023

61 24,015 53,224 541,041 0,0009

62 24,448 7503,629 73865,625 0,1284

63 25,432 473,663 7873,599 0,0137

64 25,615 4639,873 43115,461 0,0750

65 26,115 239,263 4267,137 0,0074

66 26,532 809,922 8269,367 0,0144

67 26,932 223,834 2348,606 0,0041

68 27,165 214,283 1460,259 0,0025

69 27,598 1345,490 28978,867 0,0504

70 28,532 142,573 2017,341 0,0035

71 29,115 22,400 96,100 0,0002

72 29,765 100,333 1935,282 0,0034

73 30,032 345,725 7065,617 0,0123

74 31,498 153,323 3821,700 0,0066

**CE2**

Peak No. Peak ID Ret Time Height Area Conc.

1 0,030 45,970 128,001 0,0003

2 2,945 969503,125 12158617,000 25,6774

3 3,372 4960,608 40246,004 0,0850

4 3,788 4771,937 26791,898 0,0566

5 4,117 2559,021 18945,514 0,0400

6 4,632 430,292 2366,401 0,0050

7 4,837 440,757 2442,702 0,0052

8 5,238 4465,907 112021,969 0,2366

9 5,485 3704,613 72705,547 0,1535

10 5,987 5939,635 38558,754 0,0814

11 6,842 3599,639 15459,091 0,0326

12 7,298 56,786 201,052 0,0004

13 7,528 69,746 506,352 0,0011

14 7,828 283437,000 1115848,750 2,3565

15 8,063 4520,655 24013,984 0,0507

16 8,273 1274,779 6295,608 0,0133

17 8,493 539,717 5397,483 0,0114

18 8,887 36881,457 159011,500 0,3358

19 9,215 254,399 1933,662 0,0041

20 9,403 282,923 1614,496 0,0034

21 9,547 184,274 668,748 0,0014

22 9,773 148,556 941,603 0,0020

23 10,163 969444,125 7649111,000 16,1539

24 10,687 17093,842 63151,156 0,1334

25 10,797 22480,063 95264,305 0,2012

26 11,223 27862,807 154554,078 0,3264

27 11,735 2903,719 12351,799 0,0261

28 11,933 8246,683 41344,379 0,0873

29 12,647 462781,563 4476585,000 9,4540

30 13,317 968532,000 10483252,000 22,1392

31 13,965 6573,818 40644,328 0,0858

32 14,228 362011,188 1603178,375 3,3857

33 14,980 228572,719 1067175,625 2,2537

34 15,512 93550,047 355673,875 0,7511

35 16,083 1357,328 3957,796 0,0084

36 16,252 3410,963 15069,411 0,0318

37 16,552 12175,732 50127,848 0,1059

38 17,148 3956,495 15139,399 0,0320

39 17,398 220635,672 1184756,375 2,5020

40 17,865 1513,090 15023,240 0,0317

41 18,205 673283,063 4429902,000 9,3554

42 18,760 1526,606 9343,155 0,0197

43 18,923 2621,375 12828,600 0,0271

44 19,777 22441,688 111783,156 0,2361

45 20,097 3496,678 33685,582 0,0711

46 20,538 90266,234 739385,313 1,5615

47 21,373 89753,633 646067,813 1,3644

48 21,943 817,716 10222,391 0,0216

49 22,350 1602,040 19395,703 0,0410

50 23,377 498,178 9424,860 0,0199

51 23,813 371,568 4650,873 0,0098

52 24,722 8244,297 81120,578 0,1713

53 25,915 4990,291 50032,402 0,1057

54 27,232 113,709 604,651 0,0013

55 27,467 152,979 953,000 0,0020

56 27,917 1106,078 26675,572 0,0563

57 30,332 622,891 34310,621 0,0725

**PC 1**

**Peak No. Peak ID Ret Time Height Area Conc.**

1 3,298 970477,875 9235542,000 80,6470

2 3,532 5199,000 15453,650 0,1349

3 3,657 693,200 907,300 0,0079

4 3,915 267,067 837,050 0,0073

5 4,240 1201,360 5798,900 0,0506

6 4,490 112,286 469,796 0,0041

7 4,590 250,714 779,803 0,0068

8 4,907 126,577 586,595 0,0051

9 4,998 156,113 632,512 0,0055

10 5,082 426,690 1805,384 0,0158

11 5,240 1084,887 3897,407 0,0340

12 5,515 54,859 266,969 0,0023

13 5,765 3200,113 107891,023 0,9421

14 6,432 2032,454 24675,795 0,2155

15 6,848 733,542 8718,342 0,0761

16 7,298 457,398 2762,630 0,0241

17 8,282 14607,200 52618,801 0,4595

18 8,532 276,200 1146,301 0,0100

19 8,765 332,730 1332,429 0,0116

20 9,082 264,703 1963,869 0,0171

21 9,365 2658,300 8471,199 0,0740

22 9,715 95,125 1429,199 0,0125

23 10,315 236,818 1413,118 0,0123

24 10,498 64521,816 267119,781 2,3326

25 11,148 826,909 3595,271 0,0314

26 11,265 665,182 3577,400 0,0312

27 11,665 1499,545 9865,127 0,0861

28 12,198 193,364 2375,626 0,0207

29 12,382 349,364 1334,355 0,0117

30 12,515 171,818 1689,628 0,0148

31 12,865 29068,189 135270,359 1,1812

32 13,298 743,068 5668,265 0,0495

33 13,515 77477,008 49383,496 0,4312

34 14,032 217,000 676,400 0,0059

35 14,165 1181,919 5213,275 0,0455

36 14,332 548,717 1817,401 0,0159

37 14,565 38401,434 145273,656 1,2686

38 15,048 127,847 946,149 0,0083

39 15,332 20371,502 95019,969 0,8297

40 15,815 3957,916 22910,916 0,2001

41 16,165 291,491 1260,406 0,0110

42 16,448 152,147 937,360 0,0082

43 16,665 1880,884 11985,322 0,1047

44 16,898 1094,601 7631,248 0,0666

45 17,115 168,338 1073,137 0,0094

46 17,315 401,095 2655,200 0,0232

47 17,498 808,873 4682,335 0,0409

48 17,748 17332,570 92895,969 0,8112

49 18,048 1418,205 8741,551 0,0763

50 18,198 897,023 6870,892 0,0600

51 18,482 150397,672 734219,688 6,4114

52 19,048 194,991 1021,949 0,0089

53 19,165 171,850 1028,656 0,0090

54 19,315 199,668 1114,875 0,0097

55 19,615 957,303 7578,923 0,0662

56 19,965 149,169 805,124 0,0070

57 20,198 3264,562 17105,438 0,1494

58 20,532 1121,124 11270,947 0,0984

59 20,798 6478,573 46141,906 0,4029

60 21,098 493,079 3965,380 0,0346

61 21,498 557,545 4085,862 0,0357

62 21,715 5417,000 32351,309 0,2825

63 22,415 172,692 1079,900 0,0094

64 22,782 131,585 1299,946 0,0114

65 23,032 108,170 1402,351 0,0122

66 23,798 264,618 2518,100 0,0220

67 24,332 86,538 1363,000 0,0119

68 24,798 39,947 340,200 0,0030

69 25,182 643,968 5774,700 0,0504

70 26,182 243,627 4260,046 0,0372

71 26,415 423,966 4857,167 0,0424

72 26,782 393,783 4706,671 0,0411

73 27,432 1294,369 60982,250 0,5325

74 28,415 1889,152 55493,617 0,4846

75 28,882 1556,829 58239,566 0,5086

76 30,848 424,784 18939,500 0,1654

**PC2**

Peak No. Peak ID Ret Time Height Area Conc.

1 3,940 970585,375 7391116,000 53,6815

2 5,877 61,845 194,999 0,0014

3 6,082 358,747 1074,698 0,0078

4 6,522 53,000 190,300 0,0014

5 6,625 28,196 70,351 0,0005

6 6,862 1112,958 17117,195 0,1243

7 7,385 156,750 551,198 0,0040

8 7,758 468,126 2358,494 0,0171

9 8,270 175,422 672,252 0,0049

10 8,817 126,461 393,399 0,0029

11 9,087 54,699 175,583 0,0013

12 9,262 9832,134 40955,125 0,2975

13 9,507 360,535 2351,555 0,0171

14 9,753 168,867 767,988 0,0056

15 10,015 533,005 3156,302 0,0229

16 10,337 3369,073 14874,150 0,1080

17 11,247 296,377 1552,000 0,0113

18 11,492 179554,125 746741,813 5,4236

19 12,125 1612,353 5525,315 0,0401

20 12,232 1633,612 6326,845 0,0460

21 12,505 682,406 2899,727 0,0211

22 12,597 5687,976 20761,105 0,1508

23 13,043 105,979 560,294 0,0041

24 13,125 218,991 697,442 0,0051

25 13,285 1393,406 4861,167 0,0353

26 13,403 387,896 1804,253 0,0131

27 13,735 138129,031 608563,375 4,4200

28 14,155 2706,051 20446,541 0,1485

29 14,403 372073,000 1638727,500 11,9020

30 14,925 2353,767 23942,305 0,1739

31 15,153 2806,821 12880,432 0,0936

32 15,252 1126,495 5036,037 0,0366

33 15,388 131238,656 483269,063 3,5100

34 15,832 144,175 476,842 0,0035

35 15,970 6933,689 30075,572 0,2184

36 16,123 70022,484 255767,391 1,8576

37 16,582 12270,911 57728,699 0,4193

38 16,773 847,445 3821,645 0,0278

39 16,860 513,641 2321,178 0,0169

40 16,958 727,662 3469,248 0,0252

41 17,105 331,851 1480,099 0,0107

42 17,387 2116,234 8746,193 0,0635

43 17,440 2102,719 9579,147 0,0696

44 17,697 2445,700 15925,707 0,1157

45 17,917 240,350 1102,029 0,0080

46 18,155 634,063 2072,799 0,0151

47 18,358 1652,451 7997,306 0,0581

48 18,545 10323,661 31451,197 0,2284

49 18,613 38379,609 218666,453 1,5882

50 19,110 1360,435 9950,821 0,0723

51 19,450 303577,031 1635807,250 11,8808

52 20,062 281,204 1792,303 0,0130

53 20,298 490,992 2572,346 0,0187

54 20,507 930,693 8921,677 0,0648

55 20,913 280,076 1314,796 0,0095

56 21,218 6209,932 33843,273 0,2458

57 21,557 3039,250 32332,914 0,2348

58 21,800 12307,163 106985,500 0,7770

59 22,152 1537,540 15298,940 0,1111

60 22,438 906,147 10022,862 0,0728

61 22,775 12125,995 100196,758 0,7277

62 23,042 898,656 7943,758 0,0577

63 23,540 499,149 7920,131 0,0575

64 23,880 409,348 5652,613 0,0411

65 24,172 271,556 3585,138 0,0260

66 24,728 60,208 367,876 0,0027

67 24,937 162,856 2292,838 0,0167

68 25,265 127,872 1408,279 0,0102

69 25,705 251,758 8278,341 0,0601

70 26,487 943,475 11441,351 0,0831

71 27,058 73,478 554,555 0,0040

72 27,603 403,030 6888,719 0,0500

73 27,857 625,926 8993,076 0,0653

74 28,203 224,565 2493,984 0,0181

75 29,120 273,673 2799,794 0,0203

76 29,583 146,091 1650,287 0,0120

77 29,915 122,676 1060,548 0,0077

78 30,337 957,283 24798,428 0,1801

**NEFA 1**

Peak No. Peak ID Ret Time Height Area Conc.

1 4,093 970383,750 15591107,000 69,7053

2 4,992 906,502 5160,898 0,0231

3 5,353 1703,794 6065,956 0,0271

4 5,813 630,802 1962,302 0,0088

5 6,388 212,340 140,500 0,0006

6 6,917 1961,150 31021,143 0,1387

7 7,098 2740,511 49773,910 0,2225

8 7,685 826,768 9734,889 0,0435

9 7,950 698,224 9582,256 0,0428

10 8,645 92,384 726,073 0,0032

11 8,902 15081,226 57978,887 0,2592

12 9,390 190,123 1905,745 0,0085

13 9,637 339,458 1631,984 0,0073

14 9,938 3702,123 12512,046 0,0559

15 10,460 224,910 904,549 0,0040

16 10,815 156,887 726,564 0,0032

17 11,047 322158,594 1284976,125 5,7449

18 11,658 2168,349 9769,936 0,0437

19 11,770 5190,656 27556,695 0,1232

20 12,160 13232,506 59104,469 0,2642

21 12,743 549,533 7320,333 0,0327

22 12,880 1374,002 6371,368 0,0285

23 13,000 547,348 5254,956 0,0235

24 13,410 352236,875 955653,813 4,2726

25 19,348 79113,242 1221838,750 5,4626

26 13,802 2001,611 7200,057 0,0322

27 14,040 274952,281 1093407,125 4,8884

28 14,532 1138,795 7524,109 0,0336

29 14,835 744,590 2936,612 0,0131

30 15,080 52007,613 198119,313 0,8858

31 15,747 24079,039 109471,813 0,4894

32 15,880 21788,793 90081,047 0,4027

33 16,408 10310,576 72663,719 0,3249

34 16,910 484,865 3054,743 0,0137

35 17,065 723,573 4124,144 0,0184

36 17,283 4469,569 28417,123 0,1270

37 17,610 3642,059 35891,008 0,1605

38 18,082 486,211 4818,131 0,0215

39 18,308 490,245 2971,252 0,0133

40 18,483 32763,047 266654,281 1,1922

41 18,823 1672,598 17394,160 0,0778

42 19,062 1560,685 18038,008 0,0806

43 19,348 78560,992 429885,906 1,9219

44 20,015 1150,036 9034,198 0,0404

45 20,240 852,062 10101,363 0,0452

46 20,473 1113,129 19563,334 0,0875

47 20,867 1060,924 10479,733 0,0469

48 21,170 1667,308 10523,915 0,0471

49 21,533 826,964 8718,069 0,0390

50 21,805 33021,207 288159,438 1,2883

51 22,413 803,983 8588,469 0,0384

52 22,740 22889,475 164792,453 0,7368

53 23,343 282,227 1761,961 0,0079

54 23,517 374,018 3927,321 0,0176

55 23,828 582,441 8263,028 0,0369

56 24,557 83,761 763,255 0,0034

57 24,868 150,187 2673,999 0,0120

58 25,470 40,132 204,299 0,0009

59 26,308 2054,782 23525,051 0,1052

60 27,370 272,996 3649,602 0,0163

61 27,575 916,898 11704,617 0,0523

62 28,665 367,736 3562,155 0,0159

63 29,037 145,713 1895,851 0,0085

64 29,808 996,110 23845,885 0,1066

**NEFA2**

Peak No. Peak ID Ret Time Height Area Conc.

1 4,143 970698,438 17107794,000 76,4519

2 5,143 231,699 2469,300 0,0110

3 5,602 297,482 2460,752 0,0110

4 6,118 165,730 1175,842 0,0053

5 6,330 136,757 670,439 0,0030

6 6,787 77,564 528,078 0,0024

7 7,353 2413,284 131826,438 0,5891

8 8,873 224,776 2419,223 0,0108

9 9,282 6406,864 25973,664 0,1161

10 9,975 254,496 1762,594 0,0079

11 10,282 2491,804 12852,270 0,0574

12 11,443 185829,891 1039739,688 4,6464

13 12,760 7438,281 40516,578 0,1811

14 13,687 646,406 4161,298 0,0186

15 14,330 221728,438 1652951,250 7,3868

16 15,115 147392,938 920407,313 4,1131

17 15,810 1913,779 46398,695 0,2073

18 16,373 28752,754 199512,234 0,8916

19 17,207 11513,854 96693,250 0,4321

20 17,397 12987,249 104387,844 0,4665

21 18,063 5068,745 73709,938 0,3294

22 18,865 1059,773 16036,002 0,0717

23 19,168 1539,745 26493,158 0,1184

24 19,602 1002,751 17280,672 0,0772

25 20,187 529,773 16481,801 0,0737

26 20,697 15835,753 -407762,250 -1,8222

27 21,865 36420,813 500320,906 2,2359

28 21,478 160,082 1112,032 0,0050

29 21,865 35753,570 304700,156 1,3617

30 22,710 208,215 1253,950 0,0056

31 23,227 346,375 8450,301 0,0378

32 23,952 217,448 2009,898 0,0090

33 24,472 648,124 5533,535 0,0247

34 25,303 12200,837 213127,094 0,9524

35 26,795 7957,098 120491,250 0,5385

36 27,930 167,158 3268,958 0,0146

37 28,247 261,500 3787,296 0,0169

38 28,748 238,788 4964,986 0,0222

39 29,787 693,814 47235,098 0,2111

40 30,982 1221,231 24002,895 0,1073
