# Supplementary material for: Data of added-value lipid production, Arachidonic acid, among other lipids by Mortierella elongata, using low cost simulated wastewater
Source: Data Brief. 2017 Jul 14;14:255–9. doi: 10.1016/j.dib.2017.07.015 (PMC5567393; doi:10.1016/j.dib.2017.07.015)
Supplement: Supplementary file 1 — Supplementary material [file mmc1.pdf]

Brazilian Bioethanol Science and Technology Laboratory

National Center for Research in Energy and Materials

COVER LETTER

Dear Editor of Data in Brief,

We are submitting this original data of the authors Bruna Soares Fernandes, Márcia Cristina Fernandes Messias, Patrícia de Oliveira Carvalho, Marcelo Zaiat, José Geraldo da Cruz Pradella, entitled “Data of added-value lipid production, Arachidonic acid, among other lipids by Mortierella elongata, using low cost simulated wastewater”, for evaluation.

We believe that those data is appropriate and guide to an interesting trend of bio-lipids production, using low cost carbon source.

This article presented an innovative data of feasibility to produce Arachidonic acid (ARA), as added-value Polyunsaturated Fatty Acids (PUFA) from Mortierella elongate, using simulated sugarcane vinasse as a carbon source. FAO suggests dietary supplementation with ARA for infants and adults. Since the global PUFA market is highly competitive, several companies are subsidizing the development of processes using low cost feedstock and microorganisms. M. elongata is a fungus able to accumulate large amounts of ARA using low cost carbon sources. Therefore, it was presented data of lipids production, mainly ARA, from M. elongate, using vinasse as a carbon source, never tested before. The lipids were quantified by two methods of extraction, Methanol/Chloroform (MC) and Hexane/Isopropanol (HIP) and by lipid classes. M. elongate was able to produce high amount of ARA, accumulating 26% of ARA from the total fatty acids and 156.45 mg g-1 of total TAG, a promising results comparing to other carbon sources and commercialized Mortierella oil. The data can be most explored and useful for the researcher, research student, academician and industry to acquire innovative process development and production.

All of the authors of this paper have contributed substantially to the manuscript and approved the submission of this manuscript to Data in Brief, and guarantee that there are no conflicts of interest. We declare that this is the first time we are submitting the manuscript to Data in Brief and it is not being considered for publication in another journal, book, conference proceedings, or government publication with substantial circulation.

Thank you for your attention,

Corresponding author,

Bruna Soares Fernandes

(Tel): +55 (19) 3517-5159/ +55 (19) 3512/1010

(Fax): +5519 3518-3164

(E-mail): brunasofer@hotmail.com/bruna.fernandes@bioetanol.org.br

Date
April, 2017

Postal address
10000 Giuseppe M. Scolfaro Street
Campinas - SP
Brazil

Website
http://ctbe.cnpem.br/

Telephone
+55 (19) 3512-1010

Email
brunasofer@hotmail.com
bruna.fernandes@bioetanol.org.br
